# Comparison of Nottingham Prognostic Index, PREDICT and PrognosTILs in Triple Negative Breast Cancer –a Retrospective Cohort Study

**DOI:** 10.1007/s12253-020-00846-8

**Published:** 2020-06-20

**Authors:** Anita Sejben, Tibor Nyári, Tamás Zombori, Gábor Cserni

**Affiliations:** 1 Faculty of Medicine, Department of Pathology, University of Szeged, Állomás u. 1, Szeged, 6725 Hungary; 2 Department of Medical Physics and Informatics, University of Szeged, Szeged, Hungary; 3 Bács-Kiskun Teaching County Hospital, Kecskemét, Hungary

**Keywords:** Triple negative breast cancer, Nottingham Prognostic Index, Predict, Tumor infiltrating lymphocytes, PrognosTILs, Prognosis

## Abstract

Triple-negative breast cancer (TNBC) represents a heterogenous subtype of breast cancer with generally poor prognosis. The prediction of its prognosis remains essential to clinicians in their therapeutical decision-making process. The aim of our study was to compare the validity of three multivariable analysis derived prognostic systems, the Nottingham Prognostic Index (NPI), PREDICT and PrognosTILs (a prognosticator including tumor infiltrating lymphocytes, TILs) in a series of TNBCs. Patients operated on with TNBC at the Department of Surgery, Bács-Kiskun County Teaching Hospital, Kecskemét between 2005 and 2016 were included. Clinical and pathological parameters and follow-up data were collected from medical charts. TILs were assessed retrospectively, following international recommendations. Estimated survivals of PrognosTILs, PREDICT and NPI were recorded and compared with real outcomes. Altogether 136 patients were included in this retrospective study. In univariate Cox analysis, type of surgery, pT, pN, stage, NPI and type of adjuvant therapy were the significant prognostic variables. The multivariate Cox-regression strengthened that NPI is an independent predictor of overall and disease-free survivals in TNBCs. The NPI, PREDICT and PrognosTILs could be compared directly only in a ROC curve analysis: the sensitivities and specificities of these predicting systems are rather similar with area under the curve values falling between 0.7 and 0.8, and NPI having the highest values. Our findings reflect the diverse prognosis of TNBC and highlight the difficulties of predicting its outcome. None of the three multivariable prognosticators is inferior to the others, the NPI can reliably be used for TNBCs.

## Introduction

Triple-negative breast cancer (TNBC) represents a heterogeneous subtype of breast cancer (BC) defined by the lack of immunohistochemical expression of estrogen receptor (ER), progesterone receptor (PR) and human epidermal growth factor receptor-2 (HER2), and by variable though distinct molecular profiles [1, 2]. Epidemiological data on TNBC revealed its higher prevalence among women of African ancestry, young BC patients and patients with Breast Cancer Gene-1 (BRCA-1) mutations [3]. The treatment of TNBC remains a challenge for clinicians due to its poor overall prognosis. Distant hematogenous metastasis formation and local recurrence are frequent and the treatment efficiency of TNBC is lower than in other types of BC [1, 4, 5]. By taking molecular profiles and BRCA deficiency into account, more personalized treatment methods are currently available [6]. Besides chemo- and radiotherapy, the role of immuno- and targeted therapy is increasing, both being currently under investigation with promising results [7–9].

Prediction of prognosis remains essential to clinicians in their decision-making process, helps stratifying patients by risk and better allows preparing individual treatment plans [10]. Various prognostic factors have already been presented in TNBC. Ovcaricek and coauthors described nodal status and age as independent prognostic factors for disease-free survival (DFS), whereas for overall survival (OS), only nodal status proved to be an independent factor [11]. Urru et al. have demonstrated that tumor stage at diagnosis and positive lymph node ratio are relevant predictors of survival and tumor recurrence, with the addition of Ki-67 status for recurrence prediction [12]. Asaga and coworkers have used a different approach, and analyzed clinical response to preoperative systemic chemotherapy [13].

The Nottingham Prognostic Index (NPI) was described by Haybittle and coauthors in 1982 and it was originally designed for primary operable BC. It takes tumor size, nodal stage and tumor histological grade into consideration [14]. On the basis of its equation and the values of the NPI, patients’ could be divided into three prognostic categories according to the original article: Category I (good prognosis); Category II (moderate prognosis) and Category III (poor prognosis) [14, 15]. Later the prognostic groups were subdivided to form the very good, the good, the moderate I, the moderate II, the poor and the very poor prognostic groups [16]. Different cut-off values and diverse definitions of NPI-based groups (ranging from three to ten classes) have been used by some research groups [10]. The NPI has been proven to be a valid prognostic tool in BC treatment [17].

A more complex prognostic model, PREDICT was published by Wishart and coauthors in 2010. The algorithm was developed from 5694 patients’ data from the Eastern Cancer Registration and Information Centre. The selected patients were operated on for invasive breast cancer. Based on the factors that were found to hold independent prognostic value, an algorithm was established that includes the presence of ductal carcinoma in situ (DCIS) or lobular carcinoma in situ (LCIS), age at diagnosis, menopausal state, ER, PR, HER2 and Ki-67 status, invasive tumor size, tumor grade, method of tumor detection and number of positive lymph nodes [18]. PREDICT is also endorsed by the American Joint Committee of Cancer [19]. The on line calculator estimates OS for 5, 10 and 15 years. Although the tool generally received good ratings for validity, Maishman and coauthors’ results showed that PREDICT was a great tool only in long-term survival estimates, and overestimated short-time survivals, especially in ER-positive tumors [17, 20, 21].

Tumor infiltrating lymphocytes (TILs) reflect prognosis in TNBC, since their higher proportion correlates with better outcome in this subset of breast tumors, and indicates the prominent role the immune system plays in TNBC. While TNBCs lack targeted therapy, the interest for immune modulators has increased [22, 23]. Loi and coworkers conducted a pooled analysis of 2148 patients and identified the following factors that independently influence the prognosis of primary TNBCs: percentage of stromal TILs, age, tumor size, number of positive lymph nodes, histological grade and treatment. Invasive disease-free survival (i-DFS), distant disease-free survival (d-DFS) and OS results were examined in 3 and 5 year intervals [24]. Based on the results, an equation was developed for survival estimates. For easier utilization, an online tool named PrognosTILs was developed for early stage TNBCs [25]. With this application, the 5-year and 10-year OS and DFS estimates can be calculated.

The aim of our study was to compare the validity of NPI, PREDICT and PrognosTILs in a series of TNBC cases.

## Materials and Methods

Patients operated on for histologically verified triple negative, invasive breast carcinoma at the Department of Surgery, Bács-Kiskun County Teaching Hospital, Kecskemét between 2005 and 2016 were included in our consecutive and retrospective study. Follow up data (OS and DFS) were collected from medical charts. For these outcomes, patients were followed from the date of surgical treatment until the time of recurrence or tumor-related death; those alive without recurrence and those dying from other causes were censored at the time of the last follow-up and death, respectively.

The following clinical and pathological variables were obtained for analysis: age, gender, localization, type of surgical and adjuvant treatments, histological type and grade of cancer, vascular invasion, tumor size, pT and pN categories, and stage. The NPI was calculated with the following equation: NPI = tumor size (cm) × 0.2 + nodal score (1 for pN0, 2 for pN1, 3 for pN2 or pN3) + number value from the histological grade [14]. The Nottingham Prognostic Groups were classified as excellent (EPG): ≤2.4; good (GPG): 2.41–3.4; moderate-1 (MPG1): 3.41–4.4; moderate-2 (MPG2): 4.41–5.4; poor (PGP): 5.41–6.4 and very poor (VPPG): ≥6.41 [16].

The predicted OS and DFS estimates of PrognosTILs were obtained from an online calculator [23, 25]. The estimations were based on the following parameters: age, number of positive lymph nodes, tumor size, histological grade, type of chemotherapy and proportion of stromal TILs. For the determination of the latter, the International TILs Working Group (later acting as International Immunooncology Biomarker Working Group - IIOBWG) recommendations and rules were used [25, 26]. To help in the estimation of stromal TILs, the online calibration system described by the IIOBWG and found at was also used [27, 28]. After getting accustomed with the scoring system with a hundred cases evaluated in a study by the European Working Group for Breast Screening Pathology, the calibration (etalon) pictures for different rates of stromal TILs were screensaved and printed, and these printed pictures were compared with the microscopic images displayed on a monitor for at least three areas. The mean of these estimates was rounded to the closest 10% value also allowing for 5% and 1%, with the help of the calibration picture published in the first article of the IIOBWG for the latter value [29].

The anticipated OS evaluations of PREDICT were determined with the online calculator, that required the following data: age, menopausal state, ER status, HER-2 status, Ki67 status, size of invasive tumor, grade of tumor, type of detection, number of positive lymph nodes and presence of micrometastasis in the lymph nodes [18, 19].

The Wilcoxon rank sum test was applied to analyze the correlation between recurrence or tumor-specific death and DFS or OS prediction rate of PrognosTILs and OS prediction rate of PREDICT. The OS and DFS data could not be correlated directly with the survival predictions of PrognosTILs and PREDICT, therefore the patients were classified in the following four categories: patients alive, patients who died of disease (DOD), patients alive with and without recurrence. The calculated OS and DFS survival predictions of PrognosTILs, the OS survival estimates of PREDICT and NPI scores were correlated with the 4 categories by receiver operating characteristic (ROC) curve analysis aiming to compare them and to find cut-off points. Patients DOD and patients alive categories were utilized in ROC curve analysis focusing on 5-year-OS prediction of PrognosTILs, PREDICT and NPI scores, while patients with recurrence and patients without recurrence categories were used in a ROC curve of 5-year-DFS estimates of PREDICT and NPI scores. The cut-off points identified by ROC curve analysis could show which OS and DFS rates of PrognosTILs, OS estimates of PREDICT and NPI scores are related to more frequent recurrence and tumor-specific death, respectively.

NPI was analyzed with the Kaplan-Meier method and the subgroups were compared with the log rank test. Cox-regression was utilized as univariate analysis. The parameters found significant in the univariate models were entered in a multivariable Cox proportional hazard model to identify factors of independent prognostic significance. PrognosTILs and PREDICT survival estimates could not be included in the multivariate analysis due to statistical reasons. Statistical models were fitted using SPSS Statistics V.23.0 software (IBM, SSPS 22.0, Armonk, NY USA). All statistical tests were two-sided and *p* < 0.05 values were considered statistically significant.

This retrospective study was approved by the institutional ethical committee of the Albert Szent-Györgyi Clinical Centre of the University of Szeged and the ethical committee of Bács-Kiskun County Teaching Hospital also gave a consent for the study.

## Results

Altogether, 136 patients who underwent surgical resection were included in our study. Ten patients (7.4%) were censored due to non-tumor related death. Tumor-specific death was found in 23 cases (16.9%), while 103 patients (75.7%) were alive at the last follow up, including 20 patients with recurrence (14.7%). The mean and median OS and DFS were 66.8 months and 57.5 months, 59.9 months and 41 months, respectively (range for OS: 7–170 months; range for DFS: 2–170 months). Recurrence was observed in 43 cases, including 11 cases (25.6%) with local or regional recurrence, 23 cases (53.5%) with distant metastasis and two cases with both local and distant types of recurrence. The median time to recurrence was 41 months (range: 2–170 months) Novel malignancies were found in 3 cases (7.0%; ovary [*n* = 1] and lung cancer [*n* = 2]). The median follow up was 56 months (range: 7–170 months).

The basic clinical and pathological characteristics are displayed in Table 1 [30]. The mean and median age of the patients were 59.6 and 59 years, respectively (range: 32–91). In univariate Cox-regression, the type of surgery, the pT and pN categories, the stage of the disease and the type of adjuvant therapy were found to be significant variables.

**Table 1:** Clinical and pathological characteristics of patients evaluated and the results of univariate Cox-regression [pT, pN categories defined by AJCC [27: Amin-AJCC], CMF: cyclophosphamide, methotrexate and 5-fluorouracil; second generation systemic treatment refers to anthracycline based regimens without taxanes; third generation refers to taxane containing regimens]

			pOS	pDFS
Age (years)	n	%	*p* = 0.102	*p* = 0.207
30–39	12	9.5		
40–49	15	11.9		
50–59	37	29.3		
60–69	35	27.8		
70–79	21	16.7		
80–91	6	4.8		
Laterality			*p* = 0.645	*p* = 0.958
Right	58	46.0		
Left	68	54.0		
Type of surgery			*p* = 0.354	p = 0.017
Mastectomy	24	19.0		
Breast conserving surgery	102	81.0		
Histology diagnosis			*p* = 0.626	*p* = 0.566
Carcinoma of no special type (NST)	112	88.8		
Medullary carcinoma	7	5.6		
Other	7	5.6		
Grade			*p* = 0.967	*p* = 0.88
2	5	4.0		
3	121	96.0		
pT			*p* = 0.222	*p* = 0.009
pT1	67	53.1		
pT2	55	43.7		
pT3	1	0.8		
pT4	3	2.4		
pN			*p* = 0.006	p < 0.001
pN0	75	59.6		
pN1mi	8	6.3		
pN1	31	24.6		
pN2	9	7.1		
pN3	2	1.6		
pNx	1	0.8		
Vascular invasion			*p* = 0.573	*p* = 0.400
Absent	100	79.4		
Present	26	20.6		
Stage			*p* = 0.05	p < 0.001
I	47	37.3		
II	51	40.5		
III	27	21.4		
no data	1	0.8		
Adjuvant therapy			*p* = 0.151	*p* = 0.003
Chemotherapy	10	7.9		
Radiotherapy	15	11.9		
Both	85	67.5		
Neither	16	12.7		
Generation of chemotherapy			*p* = 0.092	*p* = 0.303
Second generation	16	12.7		
Third generation	73	57.9		
Other (CMF)	6	4.8		
No data	31	24.6		

The predictions from PrognosTILs and PREDICT and the NPI scores were established in 93, 126 and 125 cases, respectively. Concerning the 5-year-OS and -DFS predictions of PrognosTILs, the mean, the median and the range of estimates are presented in Table 2. The comparison of predicted survival estimates and outcomes revealed that the predicted OS estimates of the patient DOD were significantly lower than those of patients who were alive (*p* = 0.015); similarly, the predicted DFS estimates of patients with recurrence were significantly lower, than those of patients without recurrence (*p* < 0.001). Table 3 highlights the mean, the median and the range of the 5-year-OS estimates of PREDICT. The statistical analysis strengthened, that the predicted OS estimates of patient DOD were significantly lower, than those of patients who were alive (*p* = 0.020).

**Table 2:** The 5-year overall survival (OS) and disease-free survival (DFS) predictions of PrognosTILs according to outcome. Significant differences were detected between OS predictions of patients who died of disease and patients alive, and DFS predictions of patients with and without recurrence

PrognosTILs predictions			average		median		range		Wilcoxon-test
	n	%	OS	DFS	OS	DFS	OS	DFS	pOS = 0.015
Patients deceased due to tumor	14	15.0	80.1	80.6	80	76	74–92%	69–92%	
Patients alive	79	85.0	85	82	85	83	49–95%	44–95%	
Patients with recurrence	27	29.0	80.3	77.3	80	77	49–93%	44–93%	pDFS < 0.001
Patients alive with recurrence	13	14.0	80.6	77.7	83	80	49–93%	44–93%	
Patients alive without recurrence	66	71.0	85.8	84	86	83	71–95%	67–95%	
All (where PrognosTILs was evaluated)	93	100.0	84.2	81.7	84	82	49–95%	44–95%

**Table 3:** The basic characteristics of 5-year overall survival (OS) predictions of PREDICT according to outcome. The survival estimates of patients dying of tumor progression were lower than those of patients who were alive at last follow up

PREDICT estimates						Wilcoxon-test
	n	%	mean	median	range	pOS = 0.020
Patients deceased due to tumor	23	18.3	62.9	65.5	9.2–85.1%	
Patients alive	103	81.7	71.8	78.1	7.1–86.5%	
All (where PREDICT was evaluated)	126	100	70.1	75.3	7.1–86.5%	

**Fig. 1:**
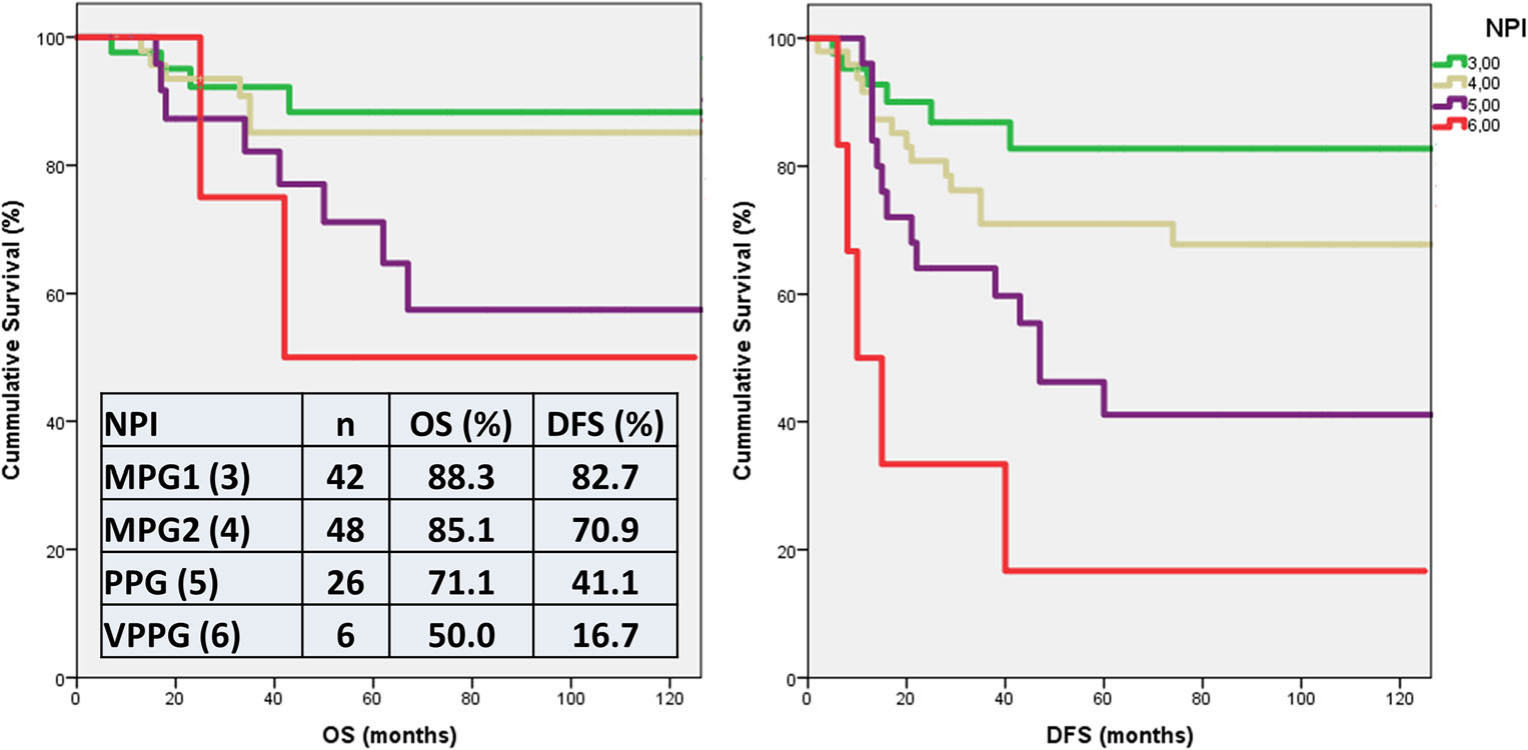
Kaplan-Meier analysis of NPI. According to the log rank test significant differences were observed between the overall survival (OS) estimates of MPG1 vs. PPG (*p* = 0.017), MPG1 vs. VPPG (*p* = 0.049) andMPG2 vs. PPG (*p* = 0.026); and the disease-free survival (DFS) estimates of PPG vs. MPG1 (*p* = 0.002), PPG vs. MPG2 (*p* = 0.035), PPG vs. VPPG (*p* = 0.013), VPPG vs. MPG1 (*p* < 0.001) and VPPG vs. MPG2 (*p* = 0.001) [MPG1: Moderate Prognostic Group 1, MPG2: Moderate Prognostic Group 2, PPG: Poor Prognostic Group, Very Poor Prognostic Group]

The NPI-based GPG included only 3 cases, therefore this group was excluded from further evaluation. Figure 1 demonstrates the results of Kaplan-Meier analysis of the NPI subgroups. Significant differences were detected between OS and DFS estimations of different prognostic groups, namely the OS estimates of MPG1 vs. PPG (*p* = 0.017), MPG1 vs. VPPG (*p* = 0.049), MPG2 vs. PPG (*p* = 0.026); and the DFS estimates of PPG vs. MPG1 (*p* = 0.002), PPG vs. MPG2 (*p* = 0.035), PPG vs. VPPG (*p* = 0.013), VPPG vs. MPG1 (*p* < 0.001) and VPPG vs. MPG2. (*p* = 0.001). In the univariate Cox-regression, NPI was found to be a significant prognostic variable (pOS = 0.022; HR:1.71, 95%CI:1.08–2.72; pDFS<0.001; HR:2.02, 95%CI:1.43–2.86).

**Fig. 2:**
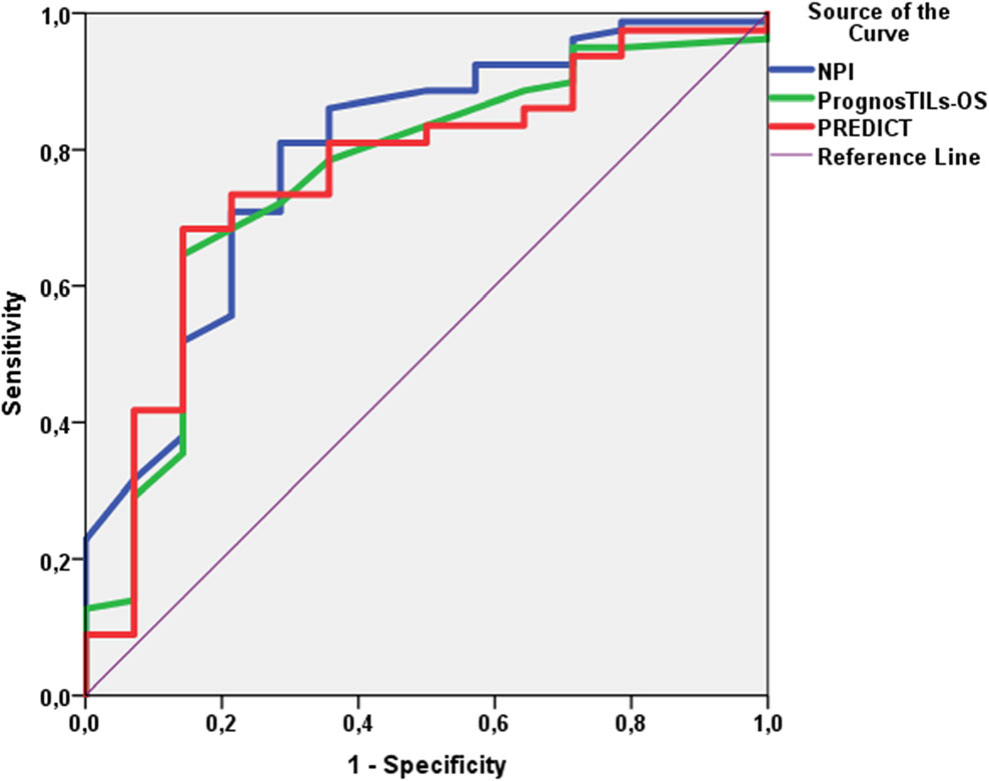
ROC curve analysis of 5-year overall survival predictions of TIL, PREDICT and NPI scores (area under the curve values for TIL, PREDICT and NPI were 0.759, 0.762 and 0.792, respectively)

**Fig. 3:**
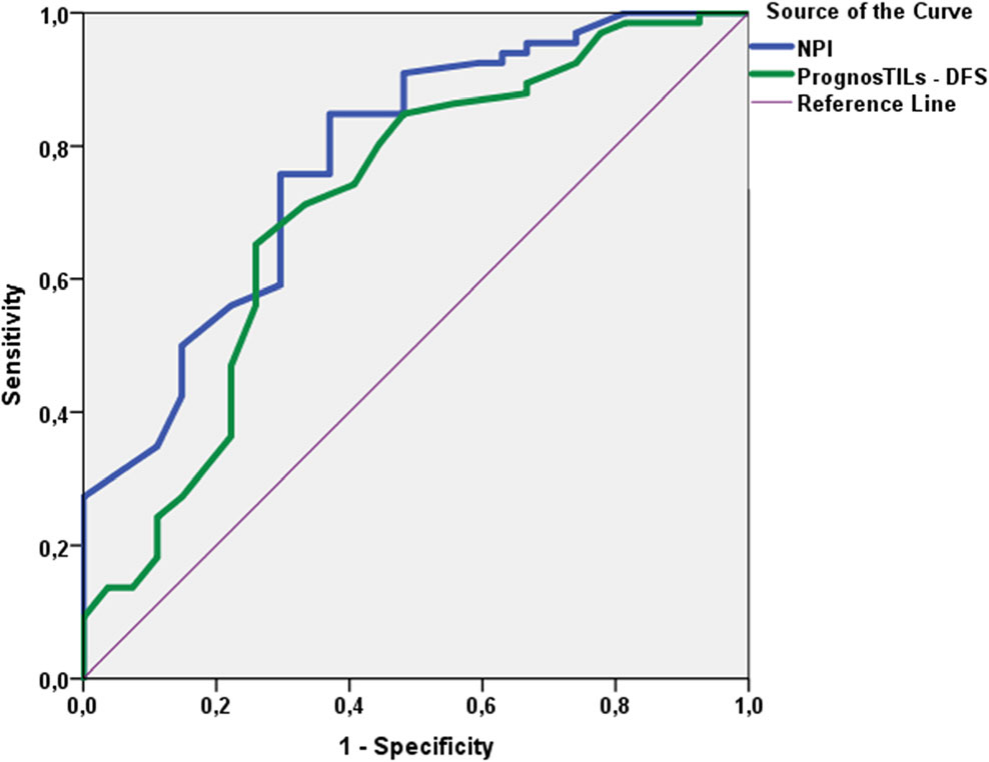
ROC curve analysis of 5-year disease-free survival predictions of PrognosTILs and NPI scores (area under the curve values for TIL and NPI were 0.713 and 0.781, respectively)

Figure 2 displays the results of ROC curve analysis focusing on 5-year-OS estimates of PrognosTILs, PREDICT and NPI scores. The area under the curve (AUC) of PrognosTILs, PREDICT and NPI were 0.759, 0.762 and 0.792, respectively. Figure 3 demonstrates the ROC curve analysis of 5-year-DFS estimates of PrognosTILs and NPI scores. The AUC values of PrognosTILs and NPI were 0.713 and 0.781, respectively. The findings of ROC curve analyses drew attention to the similarities of these predictive systems concerning sensitivity and specificity and to the fact that they are not ideal for defining cut-off values.

The multivariate Cox proportional hazard model revealed that among the variables found significant in univariate models (type of surgery, pT, pN, stage, adjuvant therapy and NPI), only NPI was an independent prognostic marker for triple negative breast cancer (pOS = 0.006; HR:1.66, 95%CI:1.16–2.37; pDFS<0.001; HR:1.92, 95%CI:1.46–2.53).

## Discussion

TNBCs are generally considered as the worst IHC based molecular subtype of breast cancer, owing to their poor prognosis and the limited therapeutic success associated with them. Despite the overall bad prognosis of TNBC, there are some tumors that by definition fall into this category, but belong to a better prognostic group. These include rare tumors like tall cell carcinoma with reversed polarity, secretory carcinoma, non-high grade, i.e. classical adenoid cystic carcinoma [31–33]. Even without these low grade special type carcinomas, the prognosis of TNBC is heterogeneous and depends on a number of prognostic factors.

The presence of distant metastasis, nodal status, tumor size and histological grade are established prognostic factors of breast carcinomas, and have their role in predicting the outcome of TNBCs as well. More recently the proportion of stromal TILs has also been recognized as an independent prognosticator of TNBCs [24], and the prognostic value of TILs was also found in a more recent meta-analysis [34]. When prognostic factors show divergent features, i.e. clinicians are faced with a combination of factors toward good and bad prognosis, predictive models based on multivariable analysis of multiple prognostic factors are much more valuable than isolated factors. The NPI is one such factor and was derived from the multivariable analysis of 387 patients with different molecular subtypes of breast cancer and was later validated in a series of 320 independent consecutive cases [35]. Several external studies have demonstrated its ability to give a prognostic classification of breast carcinomas [36–38]. Although the improvements in treatment have significantly altered the outcomes of breast cancer, and this improvement is also reflected in the NPI prognostic group-specific survivals, the prognostic separation of breast cancers on the basis of the NPI was still found to be valid [39]. The PREDICT tool was derived from a much greater population and was also independently validated in a number of reports [17, 40]. PrognosTILs is a novel multivariable prognosticator model and calculator derived from the pooled analysis of 2148 individual patients’ data from 9 studies on TNBCs proving the prognostic value of stromal TILs in the adjuvant setting [24]. This distinguishes it from NPI and PREDICT which were built on data from ER-positive and ER-negative tumors together, and theoretically could mean that it is better fitted to predict the prognosis of TNBCs.

The significance of the NPI in TNBC was first examined by Albergaria and coauthors in 2001 with reassuring results. NPI results correlated well with real survival data due to the facts that TNBCs are frequently high grade and large tumors [41]. PREDICT, to our knowledge has not yet been evaluated for TNBCs alone, whereas PrognosTILs is relatively recent for larger validation on comparison studies.

In univariate Cox analysis, type of surgery, pT, pN, stage, NPI and adjuvant therapy were found significant prognostic variables. We also found that lower 5-year OS and DFS predictions of PognosTILs are related with more frequent tumor specific death and recurrence (pOS = 0.015, pDFS<0.001), while the lower 5-year OS predictions of PREDICT are associated with higher rate of tumor specific death (*p* = 0.02). Concerning the NPI, we demonstrated that there are significant differences among OS and DFS estimates of certain prognostic groups (Fig. 1). PrognosTILs and PREDICT derived estimates of survival, as scale variables could not enter the Kaplan-Meier analysis. The direct comparison of the multivariable prognosticators was performed with ROC curve analysis. Regarding the OS follow up data, PrognosTILs, PREDICT and NPI, while regarding the DFS follow up data, PrognosTILs and NPI were compared. All three predictors of outcome reflect fair performance with areas under the ROC curves falling between 0.7 and 0.8. The sensitivity and specificity of these predicting systems are rather similar, although there seems to be a tendency for NPI values to better predict outcome on the basis of the somewhat greater AUC values. In keeping with the results of Albergaria et al., the multivariate Cox-regression strengthened that NPI is an independent predictor of OS and DFS in TNBCs (pOS = 0.006; HR:1.66, 95%CI:1.16–2.37; pDFS<0.001; HR:1.92, 95%CI:1.46–2.53) [41]. Considering that the ROC curve analysis yielded similar results for the three multivariable prognosticators studied, it can be inferred that any of these is suitable to predict the outcome of TNBCs, and none of these is inferior to the others.

The results also show that TNBCs are prognostically heterogeneous. No case was classified as of very good prognosis on the basis of the NPI, and only 3 cases fell into the good prognostic group. This is due to the fact that only 5 tumors were of histologic grade 2, whereas the remaining were high grade, and with this combination, their NPI value was immediately >4.

The lack of all prognostic markers for all cases and the fact that this was a single institution study of retrospective nature with limited number of cases are possible limitations of this work. A further limitation may be that values predicted by PrognosTILs and PREDICT, due to statistical reasons, could not be entered into the multivariate Cox-regression analysis, and could not be compared to NPI in this setting; but this drawback was compensated by the ROC curve analysis of the three prognosticators. Our study has strengths, as well. To our knowledge, this study is the first to evaluate the value of PREDICT in TNBCs, and these multivariable prognostic tools have never been compared in a single study. Another advantage of the study design was the uniform evaluation of TILs with rigorous adherence to internationally agreed guidelines.

In conclusion, our findings reflect the diverse nature of TNBC and highlight the difficulties of predicting the outcome of this disease. Although the NPI seemed to give somewhat higher AUC values in the direct comparisons with PREDICT and PrognosTILs, none of the multivariable prognosticators is inferior to the others according to our data.

## Data Availability

The datasets generated during and/or analyzed during the current study are available from the corresponding author on reasonable request.
